# The prenatal imaging of a rare congenital intracranial teratoma

**DOI:** 10.1016/j.radcr.2024.06.073

**Published:** 2024-07-20

**Authors:** Andrea Vrionis, Julia Hegert, Larry Matsumoto, Laura Hayes, Jennifer Neville Kucera

**Affiliations:** a University of South Florida Health, Morsani College of Medicine. 560 Channelside Dr, Tampa, FL 33602, USA; b Orlando Health, Department of Pathology, 92 W Miller St, Orlando, FL 32806, USA; c Sarasota Memorial Hospital, Department of Maternal Fetal Medicine. 1700 S Tamiami Trail, Sarasota, FL 34239, USA; d Nemours Children's Hospital, Department of Radiology, 6535 Nemours Pkwy, Orlando, FL 32827, USA; e University of Central Florida, Department of Radiology, 6850 Lake Nona Blvd, Orlando, FL 32827, USA; f University of South Florida, Department of Radiology, 2 Tampa General Circle, STC 6102, Tampa, FL 33606, USA

**Keywords:** Teratoma, Congenital tumor, Intracranial mass, Hydrocephalus, Macrocephaly

## Abstract

Fetal intracranial teratoma presents a rare and devastating diagnosis. Typically, this condition is first detected during routine prenatal ultrasounds, appearing as an irregular heterogeneous lesion. Further insights are gained through fetal magnetic resonance imaging (MRI), better characterizing the anomaly. The combination of these modalities provides detail-oriented high resolution MRI images, while follow-up ultrasounds capture dynamic growth changes, serving as a cost-effective and easily accessible adjunct. This fast-growing tumor leads to macrocephaly and ventriculomegaly, causing severe distortion of the brain parenchyma. Early detection is crucial for effective fetal management and preventing maternal complications. Unfortunately, treatment options are limited due to the tumor's aggressive nature, typically resulting in fetal demise shortly after birth. Here, we present the sonographic and MRI findings of a congenital intracranial teratoma, reaching massive proportions and replacing the entire cerebral hemisphere.

## Introduction

Congenital tumors are a rare occurrence, accounting for less than 1% of all pediatric malignancies [1]. Among fetal tumors originating from the central nervous system, teratomas predominate with an incidence ranging from 25% to 50% [2,3]. While these tumors commonly manifest in the sacrococcygeal region, sporadic occurrences have been documented in areas such as the orbit, gonads, and cranial cavity [4]. Intracranial teratomas portend a particularly dismal prognosis, as they not only invade the brain directly but also impede the growth of healthy tissue through mass compression. Known for their rapid growth, mortality rates run as high as 93%, with significant morbidity affecting the remaining 7% [5]. We describe a case of a massive intracranial teratoma exhibiting exponential growth in utero, ultimately precipitating fetal hydrops.

## Case report

We present a case of a 34-year-old G3P1011 referred to maternal fetal medicine for a fetal brain abnormality. She had a surgical history of a prior caesarean delivery but otherwise no significant medical history. She had negative carrier screen testing and a cell free DNA screening which indicated a low-risk profile.

**Fig. 1:**
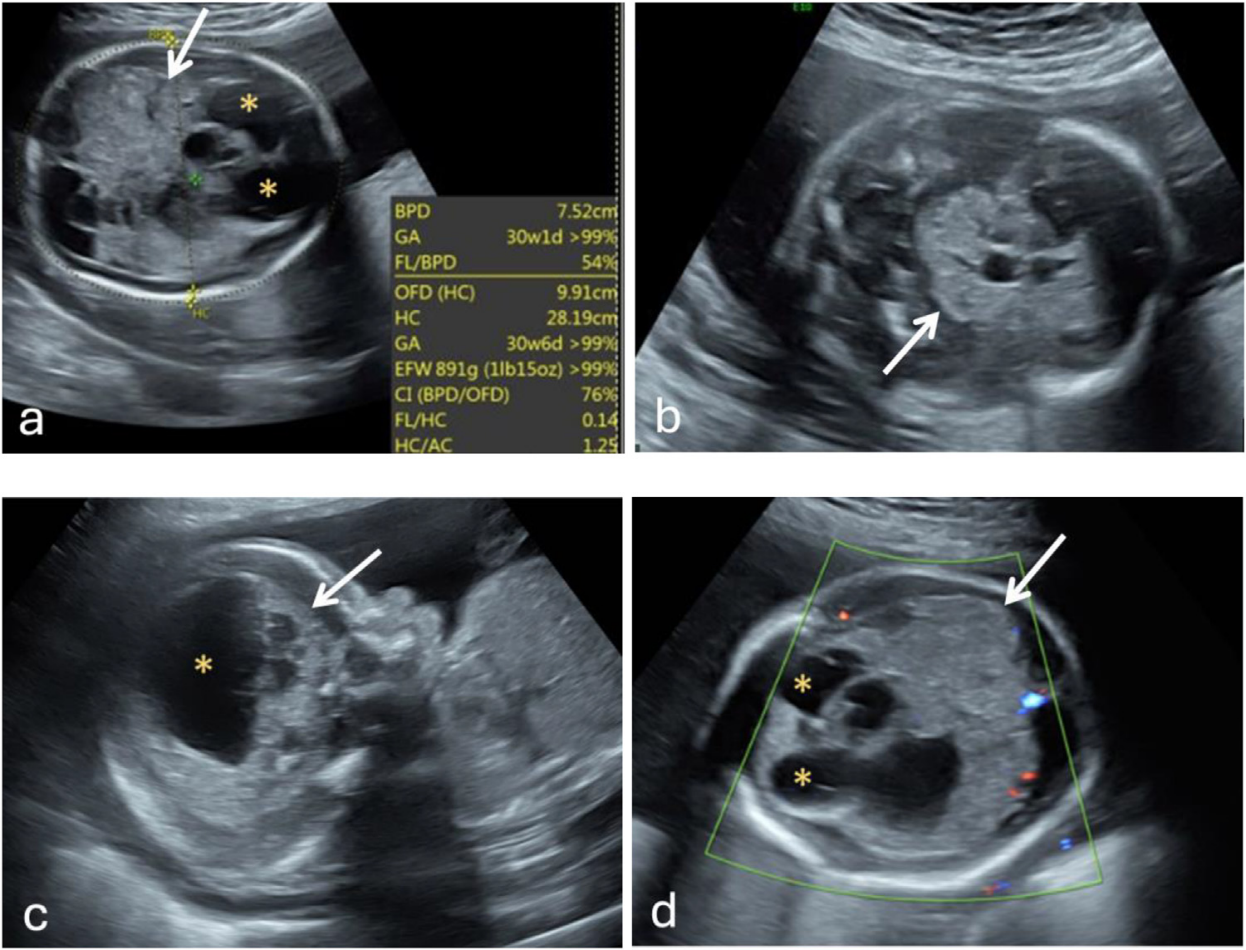
Detailed fetal anatomy ultrasound at 24 weeks and 0 days. Transverse (A, B, D) and sagittal (C) planes display architectural distortion within the cranial cavity. The supratentorial brain parenchyma has a markedly dysmorphic, heterogenous appearance (arrows) with dilated lateral ventricles (asterisks). Head circumference measurements (A) are consistent with a 30-week and 6 days gestation fetus, >99th percentile for gestational age. The relative lack of blood flow (D) in the region on 2D color flow mapping narrowed the differential to a complex evolving hemorrhage, ischemia, or mass.

**Fig. 2:**
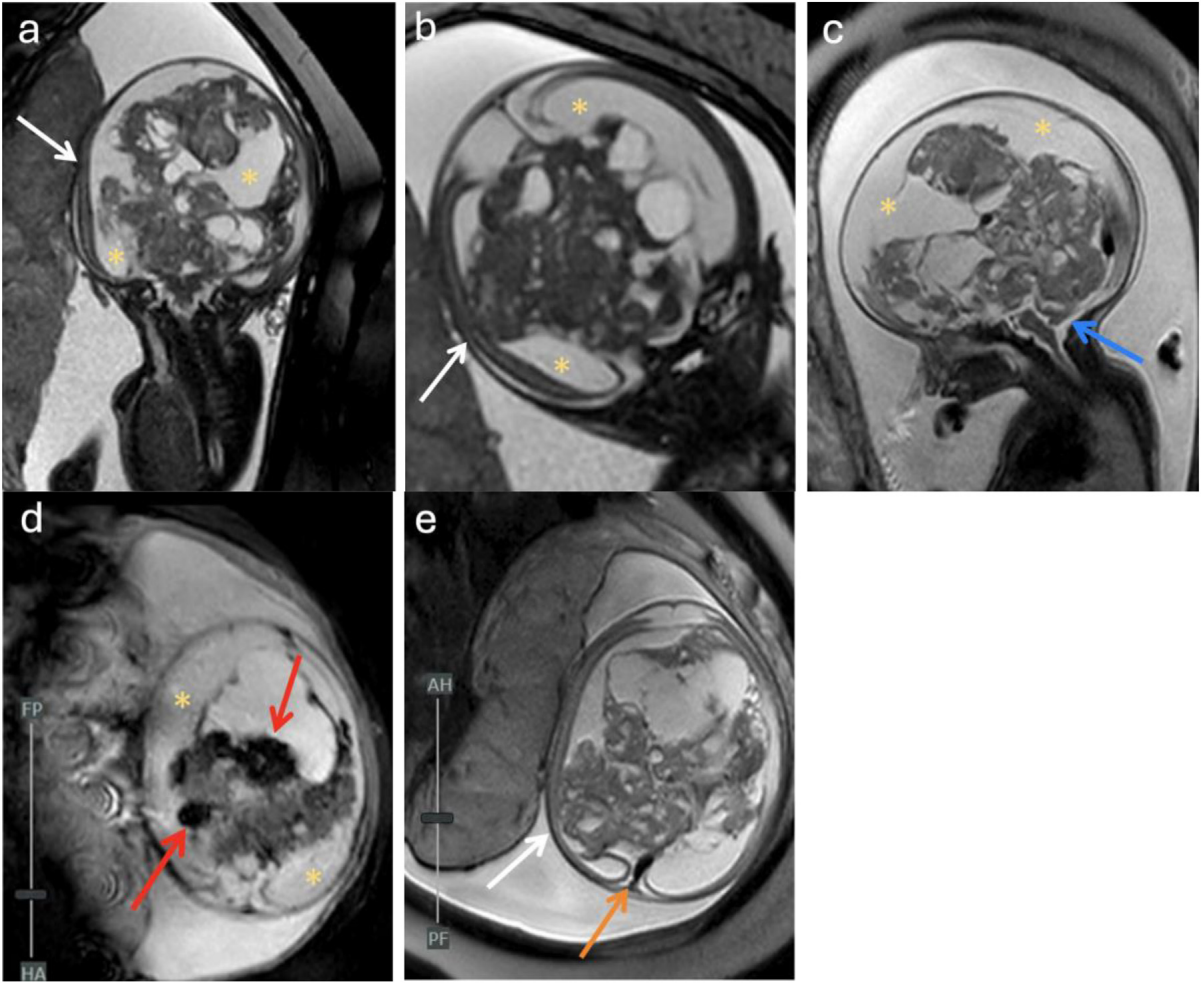
Fetal MRI performed at 25 weeks and 5 days. Coronal T2 Fiesta (A, B), sagittal T2 SSFSE (B), and axial T2 EPI Gradient (D) display solid and cystic components of the heterogenous midline supratentorial mass with dilated lateral ventricles (asterisks) and compressed brain parenchyma laterally (white arrows). Lateral ventricles measure up to 3.1 cm, left side more severe than the right. Severe hydrocephalus is observed with resulting downward displacement of the cerebellum (C, blue arrow). The disproportionally enlarged head relative to the fetal body is best visualized in the sagittal plane (C). Black areas of dark gradient signal (red arrows) scattered throughout the lesion in image D likely represent a combination of calcifications and hemorrhage within the mass. The engorgement of the sagittal sinus (E, orange arrow) suggests a highly vascular lesion or venous compression. The collective findings are consistent with congenital immature teratoma. Although the exact size of the tumor is difficult to accurately measure due to its large size and architectural distortion, it measures at least 9.5 cm anteroposterior by 8.2 cm transverse by 7.5 cm craniocaudal.

Abnormalities of the fetus were first detected on a routine prenatal ultrasound performed at 21 weeks’ gestation, noting a dilated right lateral ventricle and a “dangling” choroid plexus. A fetal anatomy ultrasound was performed at 24 weeks to further evaluate the brain abnormality, now revealed as a 6.4 × 4.1 cm mass (Fig. 1). Initially, there was a lack of blood flow observed on 2D color flow mapping, suggesting possible etiologies such as evolving ischemia or a large intracranial hemorrhage with clot. For further workup, maternal and paternal blood was tested for human platelet antigen incompatibility, resulting negative. A fetal MRI was performed at 25 weeks and 5 days for further assessment of the intracranial abnormality. The MRI revealed a large heterogeneous midline brain mass measuring 9.5 × 8.2 × 7.5 cm. This was accompanied by severe ventriculomegaly, head enlargement, and peripheral compression of brain parenchyma, and downward displacement of the cerebellum (Fig. 2). The engorgement of the sagittal sinus hinted at a highly vascular lesion or venous compression, while the areas of dark gradient signal provided further characterization of the mass, indicating possible calcifications and/or hemosiderin deposition. Collectively, these findings were consistent with congenital immature teratoma.

Just 8 days later, ultrasound measurements showed the tumor had further enlarged to 13.1 × 10.67 cm. There was also development of fetal pleural effusions, enlargement of the kidneys, and mild cardiomegaly, raising concerns for fetal high output heart failure stemming from the highly vascular lesion. After extensive discussions with parents regarding the dismal prognosis, they chose to proceed with the pregnancy with comfort care only.

**Fig. 3:**
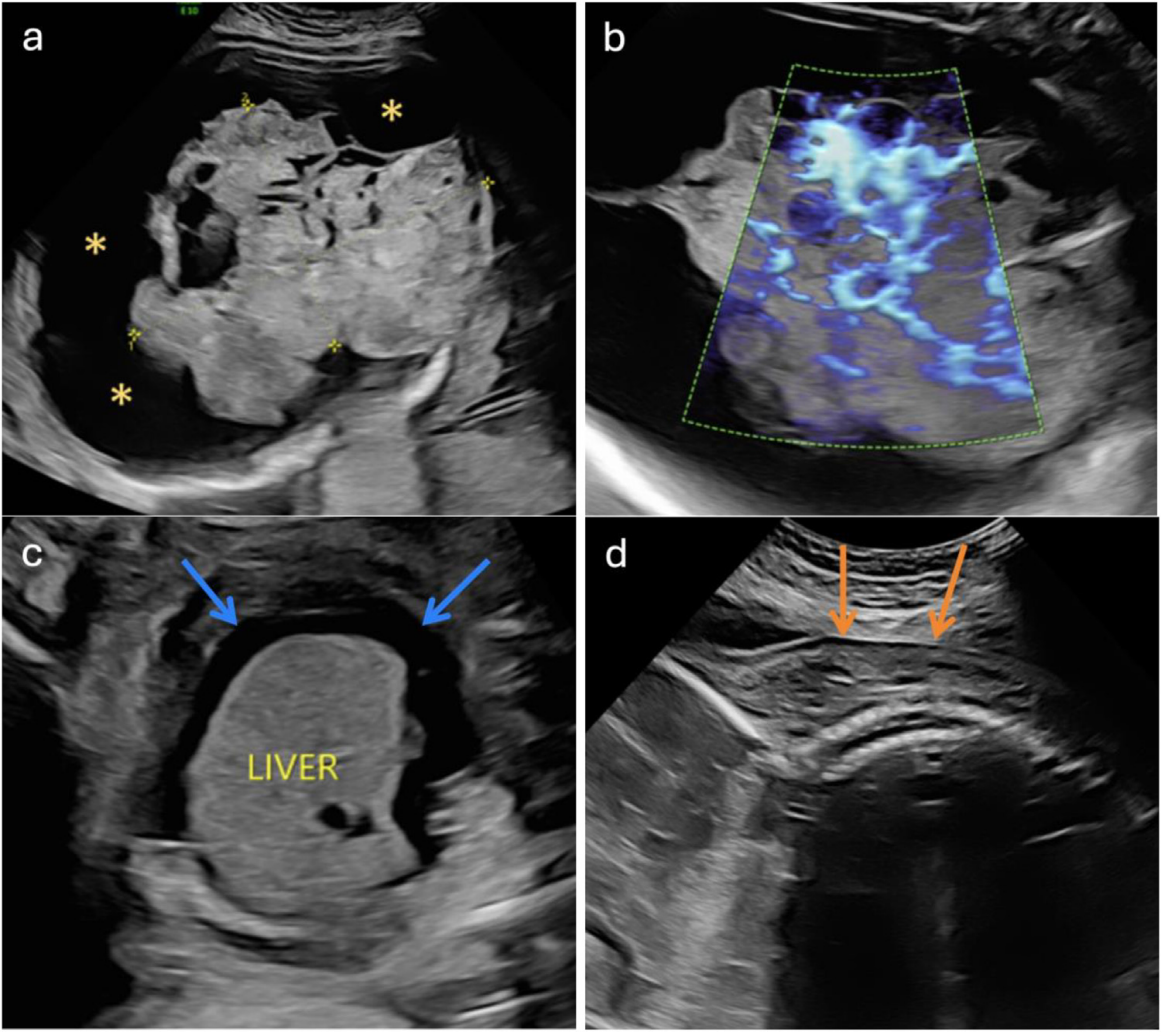
Follow-up sonogram at 29 weeks and 1 day. Transverse images demonstrate the size of the tumor, now measuring 12.59 × 8.79 cm (A), and the increase in vascularity (B), predisposing the fetus to high output cardiac failure. The cortex is severely thinned, making it difficult to visualize. There is significant fluid accumulation around the teratoma (A, asterisks). Hydrops of the fetus is observed with ascites (C, blue arrows) in the transverse plane, and skin edema (D, orange arrows) in the sagittal plane.

Follow-up ultrasound at 29 weeks and 1 day showed the development of fetal hydrops characterized by pleural effusion, ascites, and anasarca (Fig. 3). Additionally, there was further increase in both the tumor size and head circumference, along with abnormalities detected in the umbilical arterial Doppler examination.

At this time, the mother exhibited signs of preeclampsia with severe features including hypertension with a blood pressure of 153/87 mmHg, proteinuria, new-onset headache, blurry vision, lower extremity edema, exertional dyspnea, and right upper quadrant pain. Her laboratory findings revealed hypoalbuminemia at 2.1 g/dL and anemia with a hemoglobin of 8.8 g/dL and hematocrit of 27.0%. Notably, her platelet count, total bilirubin, aspartate aminotransferase, alanine aminotransferase, and alkaline phosphatase levels were all within normal ranges.

**Fig. 4:**
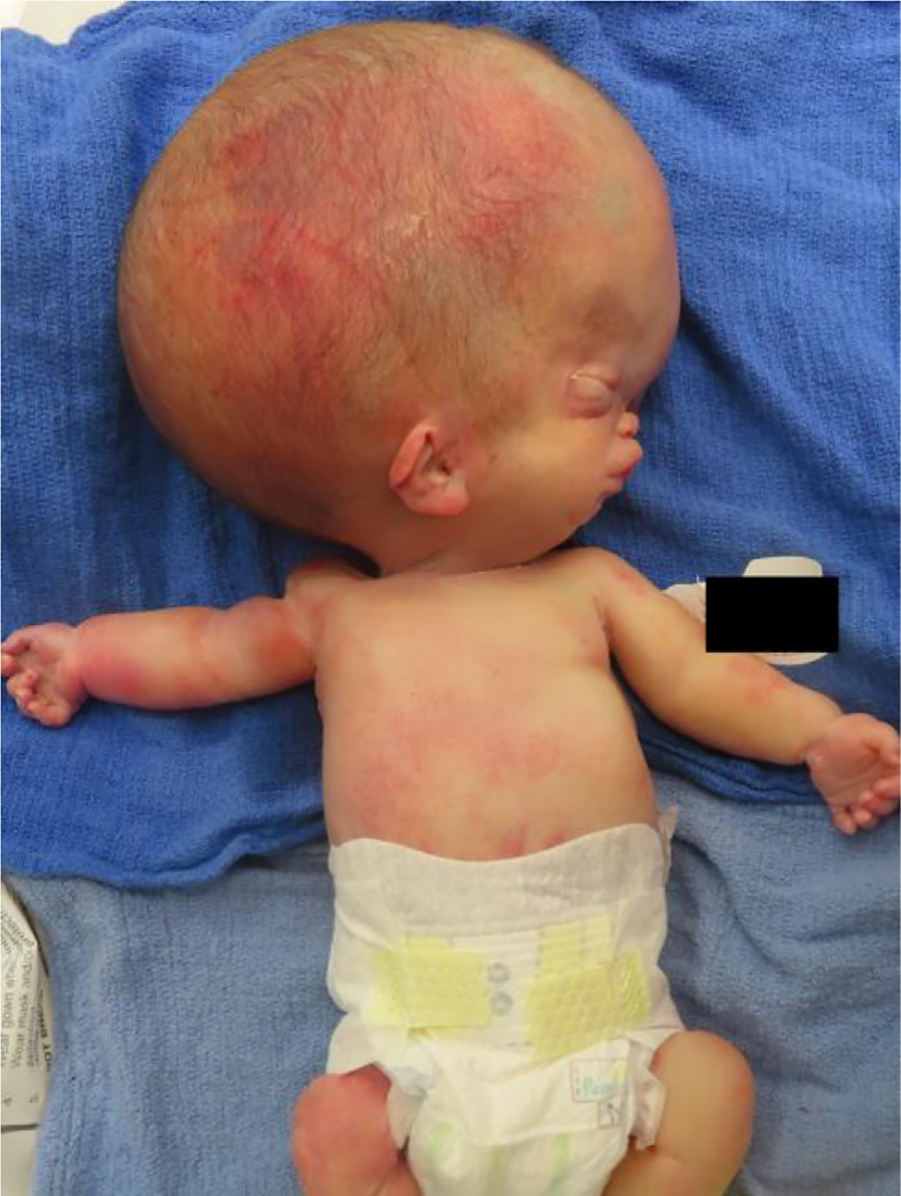
Severe macrocephaly of the male infant.

**Fig. 5:**
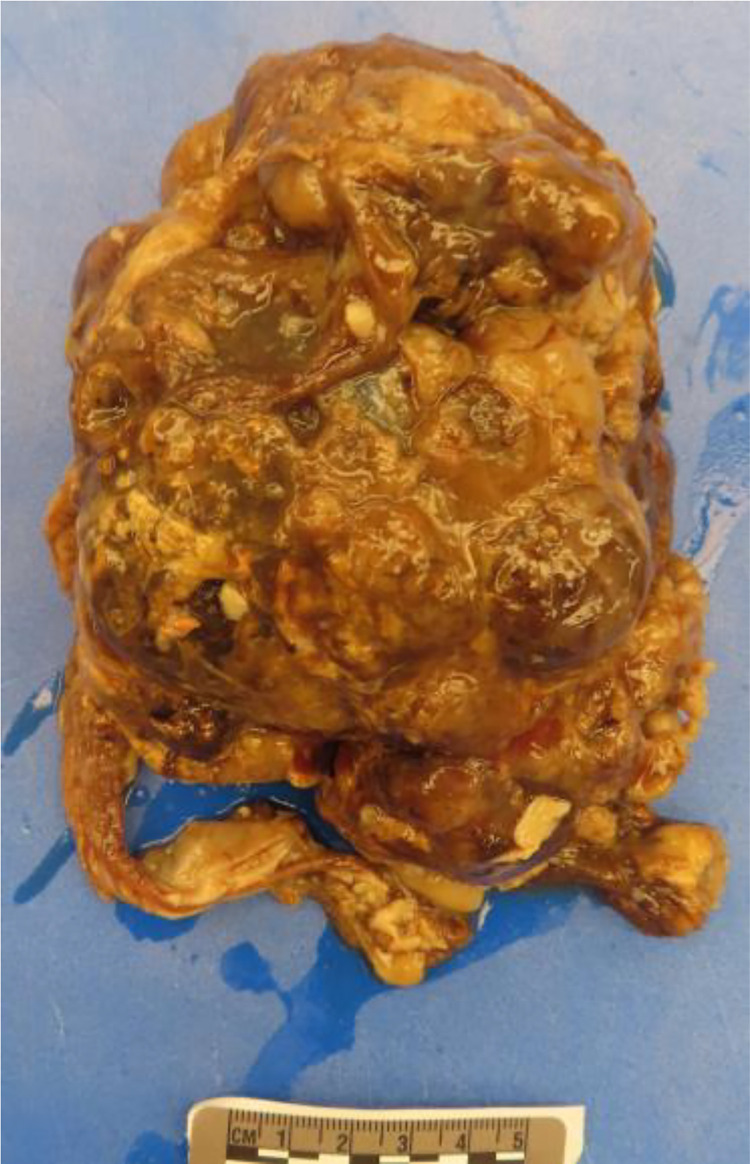
Gross specimen of the teratoma demonstrates the heterogeneity of the mass with areas of hemorrhage and calcification.

**Fig. 6:**
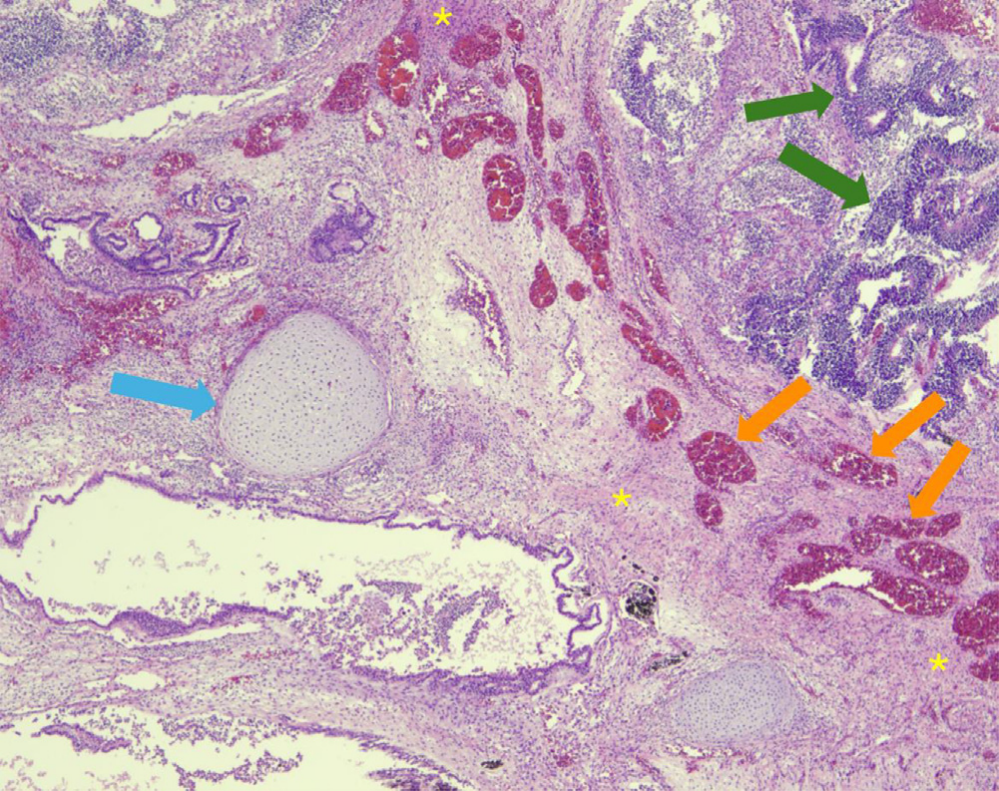
Histological examination reveals characteristics indicative of a teratoma. Endodermal elements are depicted as tubular gland-like structures (green arrows), while mesodermal components manifest as cartilage (blue arrow) and blood vessels (orange arrows). The ectodermal layer is discernible in the background as glial tissue (yellow asterisks).

The following day, at 30 weeks and 0 days gestation, the patient underwent urgent classical cesarean delivery due to concern for mirror syndrome. The male infant (Fig. 4) was delivered without complication, and the infant passed away 33 minutes after delivery. Autopsy performed the following day confirmed the presence of an intracranial teratoma, with all 3 germ layers identified both macroscopically (Fig. 5) and microscopically (Fig. 6).

## Discussion

Teratomas, a subtype of germ cell tumors, encompass tissues derived from all 3 embryonic germ cell layers: endoderm, mesoderm, and ectoderm. They can be further classified into mature teratomas, well-differentiated and benign in nature, and immature teratomas, poorly differentiated with a high likelihood of malignant transformation [6].

Intracranial germ cell tumors occur primarily in the midline region, with origins documented in the pineal gland and suprasellar region, and less commonly basal ganglia and thalamus [6]. Typically sporadic in occurrence, they have not yet been tied to any genetic syndromes [7]. Most literature hypothesizes dislocation of primordial germ cells during 3-6 weeks of gestation as the inciting factor in pathogenesis, although little is definitively known about the tumor's mechanisms [6].

The lesion is usually first detected on a routine prenatal ultrasound around 32 weeks’ gestation, although official diagnosis often comes later. The average maximum tumor size reaches 10 cm [8]. In our case, the tumor size was notably above average, measuring 14.32 cm, which likely facilitated its early identification at 24 weeks' gestation. While teratomas are among the more common congenital intracranial tumors, instances of such massive proportions with exponential growth, effectively replacing the entire cerebral hemisphere as observed in our case, are exceedingly rare [7,9,10].

MRI plays a pivotal role in further characterizing the tumor and guiding further management. The detailed images are essential for narrowing the differential, as these tumors can easily be mistaken for other more common intracranial abnormalities such as intracranial hemorrhage or ischemia, or other intracranial abnormalities [3]. Adding to the complexity of diagnosis, the specific origin of the mass within the cranial cavity is often indeterminate, due to the size of the tumor and direct brain invasion obscuring distinct anatomical landmarks. While imaging is crucial for recognition of characteristic features, the definitive diagnosis is confirmed only through histological examination after birth [11].

On both MRI and ultrasound, the tumor appears as an irregular, heterogenous mass with hyperechoic and hypoechoic regions, representing the solid and cystic components, with or without calcifications [8]. Associated features include macrocephaly and hydrocephalus, with severe distention of the head and potential for skull rupture during delivery [12]. Most infants diagnosed with intracranial teratomas before birth typically die either before or shortly after delivery. The prognosis becomes increasingly poor with younger gestational age at diagnosis and larger overall tumor size [13]. The leading causes of death in the fetus are dystocia, brain effacement by the tumor, and cranial rupture during delivery [7]. There are very few long-term survivors of congenital immature teratoma.

Despite improved recognition and earlier diagnosis in recent years, there still remain few options in terms of treatment. In utero surgery is a potential approach but carries high risks of stillbirth or premature labor [2]. Additionally, the procedure is rarely curative, and the subsequent morbidity of resection may outweigh any benefits achieved. Chemotherapy can be offered as monotherapy or an adjunct to surgery, although tumor responsivity varies and the side effects significantly impact long term quality of life [2]. Radiation therapy is generally contraindicated in neonates. As treatment options remain lacking, some suggest early offers of palliative care may offer the best quality of patient care.

These tumors also pose serious risks to maternal health. Mothers commonly present with rapidly increasing fundal height and polyhydramnios [7]. Cephalopelvic disproportion may manifest early in the pregnancy, with reports in the second trimester, leading to high caesarian section rates up to 60% [14]. Structural brain lesions, particularly vascular ones like teratomas, can trigger nonimmune hydrops in the fetus [12]. Mirror syndrome, also known as Ballantyne syndrome, describes generalized maternal edema that mirrors the edema in the fetus following the development of hydrops fetalis. A systematic literature review conducted in 2023 described the clinical presentation of mirror syndrome, including maternal edema, hypoalbuminemia, anemia, and new-onset hypertension, all of which were observed in our patient [15]. While much of the pathogenesis remains unclear, including the etiology of maternal hydrops, the study did identify certain risk factors such as fetal hydrops, alpha thalassemia, and Rh isoimmunization. Affected mothers often develop preeclampsia and pulmonary edema, with high rates of postpartum hemorrhage [16].

## Conclusion

Congenital intracranial teratomas of this size are exceptionally rare and present many challenges in terms of diagnosis, management, and treatment. Ultrasound and fetal MRI are the cornerstones of diagnosis, providing valuable information such as rate of tumor growth, degree of intracranial distortion, and identification of associated features and complications. While advancements in imaging technologies have facilitated earlier detection, treatment options remain limited, and survival beyond the immediate postnatal period is extremely rare. Management centers on counseling parents, preempting and monitoring complications, and ensuring safe and timely delivery.

## Patient consent

Written informed consent for publication of their case was obtained from the patient.
